# Microglia Implicated in Tauopathy in the Striatum of Neurodegenerative Disease Patients from Genotype to Phenotype

**DOI:** 10.3390/ijms21176047

**Published:** 2020-08-22

**Authors:** Huifangjie Li, William C. Knight, Pengfei Yang, Yingqiu Guo, Joel S. Perlmutter, John C. Morris, Randall J. Bateman, Tammie L. S. Benzinger, Jinbin Xu

**Affiliations:** 1 Department of Radiology, Washington University School of Medicine, St. Louis, MO 63110, USA; huifangjie.li@wustl.edu (H.L.); knight.w@wustl.edu (W.C.K.); pengfeiyang@wustl.edu (P.Y.); Yingqiu.guo@duke.edu (Y.G.); perlmutterjoel@wustl.edu (J.S.P.); benzingert@wustl.edu (T.L.S.B.); 2 Department of Neurology, Washington University School of Medicine, St. Louis, MO 63110, USA; jcmorris@wustl.edu (J.C.M.); batemanr@wustl.edu (R.J.B.); 3 Department of Neuroscience, Washington University School of Medicine, St. Louis, MO 63110, USA; 4 Department of Physical Therapy, Washington University School of Medicine, St. Louis, MO 63110, USA; 5 Department of Occupational Therapy, Washington University School of Medicine, St. Louis, MO 63110, USA

**Keywords:** microglia, tauopathy, striatum, oxidative damage, lewy body diseases

## Abstract

We found interactions between dopamine and oxidative damage in the striatum involved in advanced neurodegeneration, which probably change the microglial phenotype. We observed possible microglia dystrophy in the striatum of neurodegenerative brains. To investigate the interactions between oxidative damage and microglial phenotype, we quantified myeloperoxidase (MPO), poly (ADP-Ribose) (PAR), and triggering receptors expressed on myeloid cell 2 (TREM2) using enzyme-linked immunosorbent assay (ELISA). To test the correlations of microglia dystrophy and tauopathy, we quantified translocator protein (TSPO) and tau fibrils using autoradiography. We chose the caudate and putamen of Lewy body diseases (LBDs) (Parkinson’s disease, Parkinson’s disease dementia, and Dementia with Lewy body), Alzheimer’s disease (AD), and control brains and genotyped for *TSPO*, *TREM2*, and *bridging integrator 1* (*BIN1*) genes using single nucleotide polymorphisms (SNP) assays. *TREM2* gene variants were absent across all samples. However, associations between *TSPO* and *BIN1* gene polymorphisms and TSPO, MPO, TREM2, and PAR level variations were found. PAR levels reduced significantly in the caudate of LBDs. TSPO density and tau fibrils decreased remarkably in the striatum of LBDs but increased in AD. Oxidative damage, induced by misfolded tau proteins and dopamine metabolism, causes microglia dystrophy or senescence during the late stage of LBDs. Consequently, microglia dysfunction conversely reduces tau propagation. The G allele of the *BIN1* gene is a potential risk factor for tauopathy.

## 1. Introduction

The accumulation of misfolded proteins and various neuroinflammatory processes has been linked to the pathogenesis of several neurodegenerative diseases. In humans, the *MAPT* (microtubule-associated protein tau) gene, located on chromosome 17, encodes tau protein, and is crucial for cytoskeletal stability and assembly. However, hyperphosphorylation of tau leads to the formation of neurofibrillary tangles (NFTs) [1,2]. A hallmark of Alzheimer’s disease (AD) pathology is a multitude of senile plaques distributed throughout diverse brain areas, including the caudate nucleus, putamen, and ventral striatum. This hallmark has been observed in sporadic and familial cases of AD [3,4]. The relatively low frequency of striatal tauopathy observed in postmortem studies of Parkinson’s disease (PD) brains is possible because tau accumulation is limited to the entorhinal cortex and medial temporal lobe, that is, tauopathy in PD brains may have a defined distribution pattern [5,6]. Recent studies indicate that microglia are associated with tau pathology spread in a mouse model overexpressing mutant human-tau, and are involved in tau pathology propagation through the brain [7,8].

Microglia represents the chief immune defense of the central nervous system (CNS), and alterations in the microglial phenotype contribute to brain diseases [9,10]. In the 1-Methyl-4-phenyl-1,2,3,6-tetrahydropyridine (MPTP) mouse model of PD, MPTP exposure leads to neurotoxic insults that result in the activation of extracellular signal-regulated kinase (ERK) in the microglia exclusively and not in the astrocytes of the striatum or substantia nigra pars compacta (SNc) [11]. Chronic activation of microglia promotes neuron death and neurodegeneration through the increased secretion of inflammatory molecules and cytokines [12]. Coincidently, our previous research with a different patient cohort shows that a significant reduction in 18-kDa translocator protein (TSPO) density—a marker of brain microglia activation—is observed in the SNc of AD cases and dementia with Lewy bodies (DLB) cases compared with age-matched controls [13]. TSPO locates on outer mitochondrial membranes in microglia [14]. Thus, possible microglia dystrophy or loss in the late-stage of these neurodegenerative diseases could be implicated. Further, dystrophic microglia from the striatum of AD and PD brains were visualized in our immunohistochemistry study that displayed spheroidal swellings and fragmentation processes [15]. Microglia rapidly respond to pathological changes, switching into activated states, and introducing reactive oxygen species (ROS) [16]. It has been suggested that highly reactive radicals may cause damage to microglia [17,18]. 

We found that significant interactions between dopamine and oxidative damage in the striatum are involved in the late-stage of neurodegenerative diseases [19]. ROS’s toxicity is reinforced by the presence of myeloperoxidase (MPO). This enzyme catalyzes the reaction between hydrogen peroxide and a chloride ion yielding hypochlorous acid (HOCl) [20]. Microglia in the neurodegenerative disease brains are positive for MPO, which is particularly increased in microglia, neurons, and β-amyloid (Aβ) plaques and not expressed in quiescent microglia in healthy brains [21,22]. It has been reported that HOCl (30–50 μmol/L) could promote caspase-3 activation, poly (ADP-ribose) polymerase (PARP) degradation, and DNA fragmentation in endothelial cells, indicating there was an involvement of MPO-mediated oxidant [23]. Additionally, PARP1 speeds up inflammatory cytokine secretion in microglia by increasing promoter DNA accessibility via histone ADP-ribosylation [24]. Triggering receptor expressed on myeloid cell 2 (TREM2), specifically expressed by microglia in the brain, modulates microglia to recognize cellular remnants from apoptosis or misfolded proteins [10]. After activation, microglia could polarize into classic inflammatory M1 and immunosuppressive M2 phenotype through regulation by TREM2 [25]. Dysfunctional microglia in frontotemporal dementia (FTD) mouse model might be due to homozygous *TREM2* missense mutations such as p.T66M [26]. A rare missense mutation (rs75932628, p. R47H) of the *TREM2* gene has been reported as an essential risk factor for AD, FTD, and PD [27,28]. Furthermore, bridging integrator 1 (BIN1) could contribute to tau pathology progression by regulating tau clearance and promoting the release of tau enriched extracellular vesicles by microglia [29]. Single nucleotide polymorphisms (SNPs) in the *BIN1* gene, including rs744373 and rs7561528, have been identified to be significantly associated with AD in East Asian and Caucasian populations [30,31,32]. Although SNPs from BIN1 (rs744373) loci may not play a major role in PD, PD dementia (PDD), or PD-mild cognitive impairment in a Chinese population, SNPs from BIN1 (rs6733839) loci was identified in patients with Lewy body diseases (LBDs) in a Caucasian population [33,34]. As alluded to above, it is still unclear if the altered microglial function is a cause, consequence, or contribution to tau pathology. To further test this, this study investigated TSPO density and tau fibril in the caudate and putamen of postmortem human brains using quantitative autoradiography in Lewy body diseases (LBDs including PD, PDD, and DLB) and AD brains and compared the results to control groups. Sample concentrations of MPO, poly (ADP-ribose) (PAR), and TREM2 were determined via enzyme-linked immunosorbent assay (ELISA). Additionally, all samples were genotyped for *TSPO*, *TREM2*, and *BIN1* using SNP Assays. Our findings show potential interactions between genotypes and abnormal neurological phenotypes and provide a deep understanding of the microglial function implicated in tauopathy in the striatum of AD and LBDs.

## 2. Results

### 2.1. Baseline Information and Clinical Features of Study Subjects

In the present study, we collected human brains at autopsy between 3 and 47 hrs postmortem. Table 1 summarizes the baseline information and clinical features of the study participants. There were no significant differences in age at death, PMI, brain weight, onset, and disease progression, indicating that our results were not affected by these factors. There was a significant difference between the AD and control groups in the Braak NFT stage factors.

### 2.2. TREM2 (rs75932628), TSPO (rs6971), and BIN1 (rs7561528) Genotypes of the Study Subjects

In total, as shown in Table 2, we identified the *TREM2* (rs75932628), *TSPO* (rs6971), and *BIN1* (rs7561528) genotypes of 65 subjects (10 PD, 8 PDD, 10 DLB, 27 AD, and 10 age-matched controls). Although rs75932628 in *TREM2* was believed to be the risk for Alzheimer’s and Parkinson’s disease [28,35], this specific variant of the *TREM2* gene was not identified in the disease or control groups. The frequency of disease groups that carry the *TSPO* (rs6971) functional polymorphism (AG genotype) was higher than that of the control group. The highest frequency of the AG genotype was found in PDD patients. Additionally, a lower frequency of the conserved/ancestral variant (GG) was observed in the disease groups than controls, especially in PDD. The controls were split fairly among the *BIN1* gene SNP (GG) and (AG), that is 50% per allele. The highest ratio of GG carriers was found in DLB, and the highest ratio of AG carriers was found in PD when compared to controls. However, the small sample sizes limited the ability to detect significant differences in the distributions of the *TSPO* and *BIN1* polymorphisms. 

### 2.3. Myeloperoxidase (MPO) Levels in the Caudate and Putamen of the Different Groups

**Figure 1:**
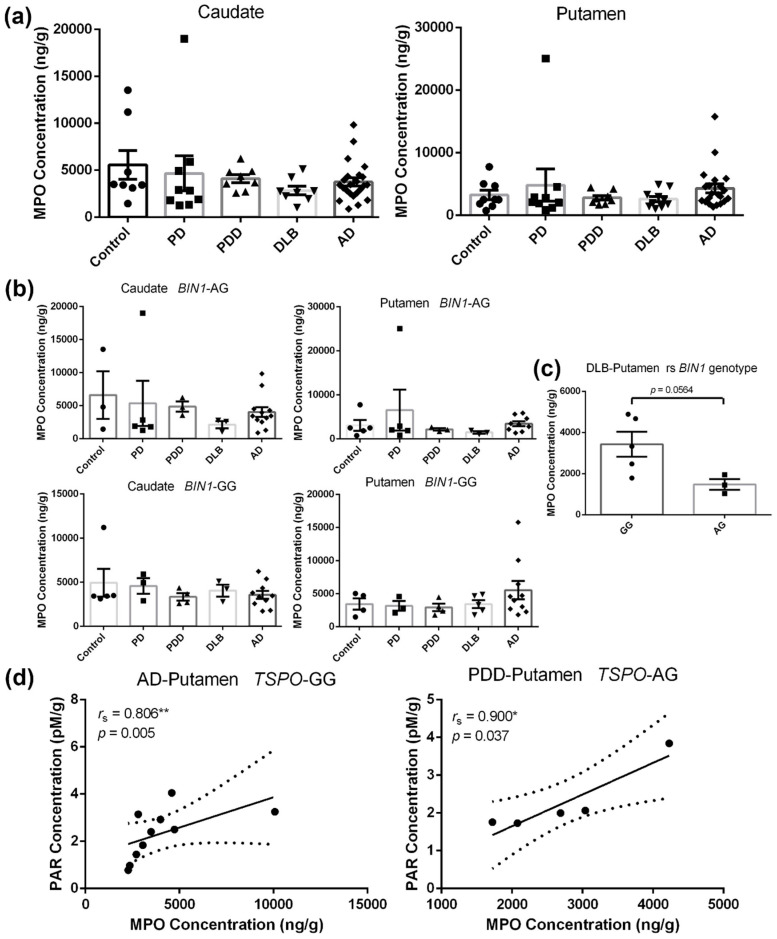
Myeloperoxidase (MPO) levels in the caudate and putamen from patients in disease groups (PD: *n* = 10, PD dementia (PDD): *n* = 8, DLB: n = 10, Alzheimer’s disease (AD): *n =* 27) and age-matched controls (*n =* 10). (**a**): Quantitative analysis of the concentrations of MPO in the caudate and putamen from subjects. Values shown are means ± SEM. (**b**): Concentrations of MPO in the caudate and putamen from *BIN1*-AG and *BIN1*-GG carriers. Values shown are means ± SEM. (**c**): The *BIN1* rs7561528 SNP impact the concentrations of MPO in the putamen of DLB patients, *p* = 0.0564. Values shown are means ± SEM. (**d**): Correlations of MPO vs. Poly (ADP-ribose) (PAR). *r*_s_, the Spearman’s rank correlation coefficient. A *p*-value of < 0.05 was considered significant: * indicates *p* < 0.05, ** indicates *p* < 0.01.

We performed an ELISA assay following the protocol mentioned in the methods section to ascertain MPO levels in the caudate and putamen of the disease groups and age-matched controls. Surprisingly, as shown in Figure 1a, the quantification of brain MPO levels reveals a substantial overlap between disease and healthy control subjects. However, no statistical significance was found across different groups. 

When stratified by *BIN1* (rs7561528) genotype (Figure 1b,c), the G allele on putamen MPO levels may increase the risk of DLB for patients. Patients who carried *BIN1* with homogenous GG showed a 132.5% increase in MPO levels compared to heterozygous AG carriers (*p* = 0.0564). One outlying sample found in the PD group was identified as an AG carrier (Figure 1b), and it contained the highest MPO levels of all the samples for both the caudate and putamen. 

Spearman analyses revealed significant positive correlations between MPO and PAR concentrations in the putamen of both AD patients with the *TSPO* (rs6971) GG genotype (*r*_s_ = 0.806, *p* = 0.005) and PDD patients with the *TSPO* (rs6971) AG genotype (*r*_s_ = 0.900, *p* = 0.037) (Figure 1d). The above findings provide further evidence that poly (ADP-ribose) synthesis is induced by H_2_O_2_ and modulated by MPO [36].

### 2.4. Poly (ADP-ribose) (PAR) Levels in the Caudate and Putamen of the Different Groups

**Figure 2:**
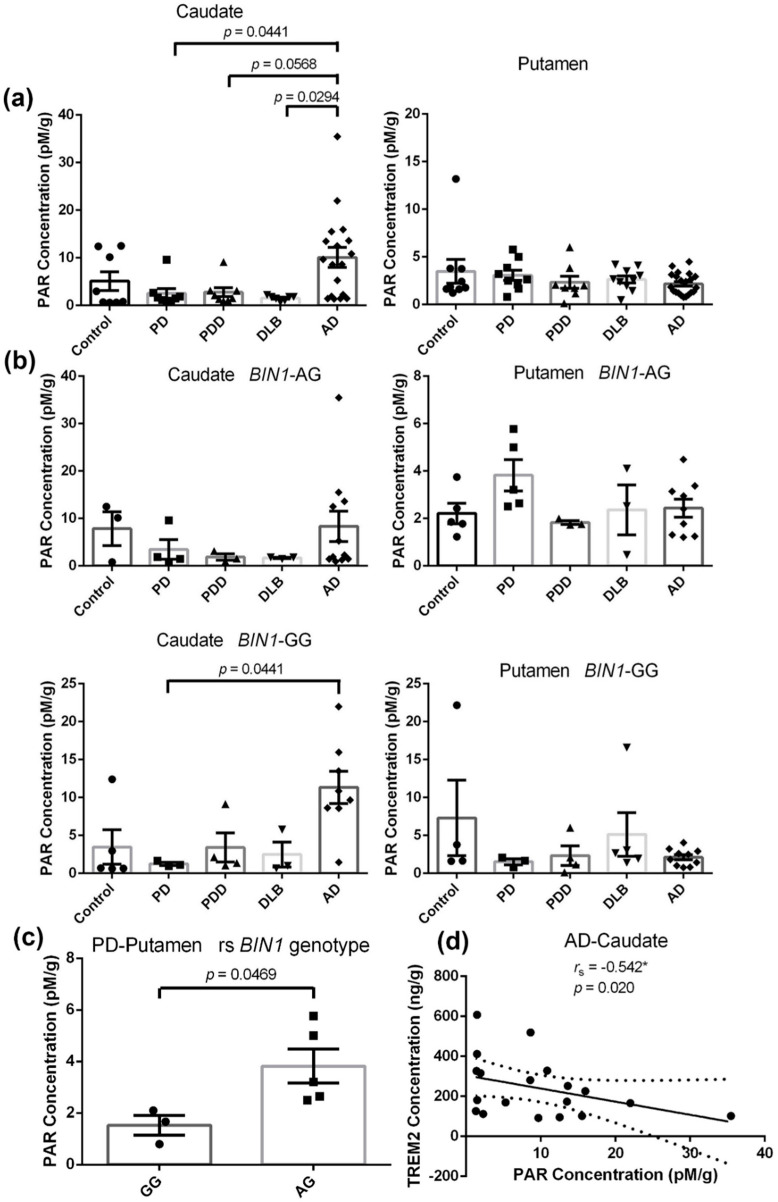
PAR levels in the caudate and putamen from patients in disease groups (PD: *n =* 10, PDD: *n =* 8, DLB: *n =* 10, AD: *n =* 27) and age-matched controls (*n =* 10). (**a**): Quantitative analysis of the concentrations of PAR in the caudate and putamen from subjects. Values shown are means ± SEM. Statistical significances between two disease groups are indicated with brackets and corresponding *p*-values. (**b**): Concentrations of PAR in the caudate and putamen from *BIN1*-AG and *BIN1*-GG carriers. Values shown are means ± SEM. Statistical significance between two disease groups is indicated with brackets and corresponding *p*-values. (**c**): The *BIN1* rs7561528 SNP impact the concentrations of PAR in the putamen of PD patients. Values shown are means ± SEM. Statistical significance between two disease groups is indicated with brackets and corresponding *p*-values. (**d**): Correlations of PAR vs. TREM2. *r*_s_, the Spearman’s rank correlation coefficient. A *p*-value of < 0.05 was considered significant: * indicates *p* < 0.05.

An ELISA assay was performed to determine the PAR concentrations in the caudate and putamen samples taken from the disease groups and age-matched controls, as shown in Figure 2a. We found that the levels of PAR in the caudate of LBDs decreased significantly compared to AD cases (PD: −75%, *p* = 0.0441; PDD: −72.3%, *p* = 0.0568; DLB: −84.8%, *p* = 0.0294). The results in the putamen vastly differed from the caudate. In the putamen, the levels of PAR in disease groups were comparable to the controls except for a small non-significant reduction in AD cases (Control: 3.475 ± 1.253; PD: 3.064 ± 0.5305; PDD: 2.336 ± 0.639; DLB: 2.627 ± 0.3691; AD: 2.166 ± 0.2415). Similar changes in PAR concentrations in the caudate and putamen are in line with our previous 8-oxo-dG levels with equivalent samples taken from the same study subjects [19].

The discovery that the rs7561528 SNP in the *BIN1* gene is associated with significantly variable PAR levels in the PD groups has provided us with a better understanding of relevant biological events. The allele G of the *BIN1* gene shows a region-specific impact on PAR variance, as shown in Figure 2b. The highest putamen PAR concentration was found in the PD patients carrying the heterozygous AG genotype (Control: 2.208 ± 0.4305; PD: 3.825 ± 0.6595; PDD: 1.828 ± 0.0846; DLB: 2.362 ± 1.053; AD: 2.434 ± 0.3816). Conversely, the lowest putamen PAR concentration was found in the PD patients carrying the homozygous GG genotype (Control: 7.306 ± 4.984; PD: 1.524 ± 0.383; PDD: 2.339 ± 1.284; DLB: 5.137 ± 2.876; AD: 2.125 ± 0.3636). Compared to AG carriers in the PD group, the level of PAR in the putamen was significantly decreased by 60.2% for these homozygous GG carriers (*p* = 0.0469) (Figure 2c). 

Additionally, the concentration of PAR showed a significant positive correlation with TREM2 expression in the caudate of AD brains (Figure 2d, *r*_s_ = −0.542, *p* = 0.020), which potentially explained the hypothesis that poly (ADP-ribose) polymerase-1 (PARP-1) modulate microglial phenotypes [37].

### 2.5. Triggering Receptor Expressed on Myeloid Cell 2 (TREM2) Expressions in the Caudate and Putamen of the Different Groups

**Figure 3:**
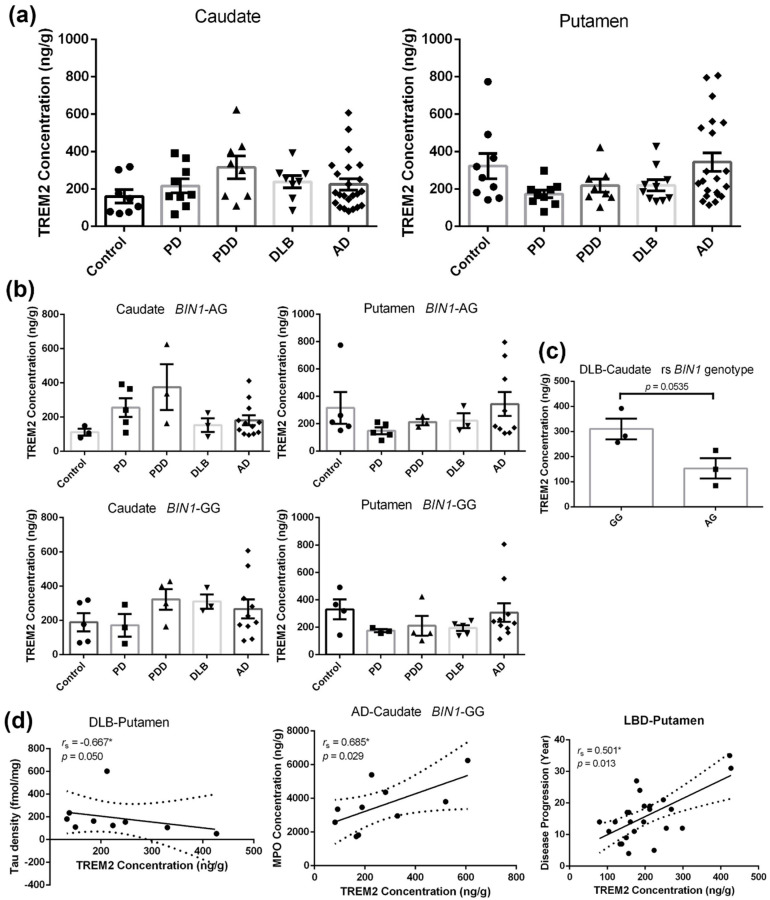
TREM2 levels in the caudate and putamen from patients in disease groups (PD: *n =* 10, PDD: *n =* 8, DLB: *n =* 10, AD: *n =* 27) and age-matched controls (*n =* 10). (**a**): Quantitative analysis of the concentrations of TREM2 in the caudate and putamen from subjects. Values shown are means ± SEM. (**b**): Concentrations of TREM2 in the caudate and putamen from BIN1-AG and BIN1-GG carriers. Values shown are means ± SEM. (**c**): The BIN1 rs7561528 SNP impact the concentrations of TREM2 in the caudate of DLB patients, *p* = 0.0535. Values shown are means ± SEM. (**d**): Correlations of TREM2 vs. Tau, TREM2 vs. MPO, and TREM2 vs. Disease Progression. *r*_s_, the Spearman’s rank correlation coefficient. A *p*-value of < 0.05 was considered significant: * indicates *p* < 0.05.

TREM2 is a type I transmembrane receptor uniquely expressed on the microglial membrane [38,39]. We quantitatively assessed the striatal TREM2 expressions of the disease groups and age-matched controls using ELISA assay, as shown in Figure 3a. TREM2 levels in the caudate and putamen of disease patients and controls did not significantly differ due to large natural variabilities in each group. We observed trends of increasing and decreasing TREM2 expressions in the caudate and putamen of the LBDs, respectively. 

When the data were stratified by *BIN1* (rs7561528) genotypes compared to the controls, we found a meaningful effect of the allele G of the *BIN1* gene on TREM2 expressions in caudate of the DLB patients (Figure 3b). Individuals carrying homozygous GG showed higher TREM2 levels than heterozygous AG carriers (Figure 3c, increased by 102.5%, *p* = 0.0535). 

Looking at the correlation between the concentration of TREM2 and tau fibrils more closely, we see a significant negative association between TREM2 expression and tau fibrils in the putamen of patients with DLB (*r*_s_ = −0.667, *p* = 0.050), as shown in Figure 3d. Changes in microglial phenotypes might help to provide a better understanding of this interaction. Additionally, the concentration of TREM2 showed a significant positive correlation with the MPO level in the caudate of AD patients identified as rs *BIN1* GG genotype (*r*_s_ = 0.685, *p* = 0.029), which is in keeping with the hypothesis that TREM2 is involved in increased neutrophil infiltration [40]. Surprisingly, TREM2 levels in the putamen of LBDs were found to increase with the disease progression (Figure 3d, *r*_s_ = 0.501, *p* = 0.013). The correlations between disease progression and TREM2 level in the putamen of PDD patients (Figure S3, *r*_s_ = 0.786, *p* = 0.036), as well PD stage and TREM2 level in the putamen of LBDs (Figure S4, *r*_s_ = 0.421, *p* = 0.036) further supported the association between microglial phenotype and disease progression [41].

### 2.6. 18-kDa Translocator Protein (TSPO) Density in the Caudate and Putamen of the Different Groups

**Figure 4:**
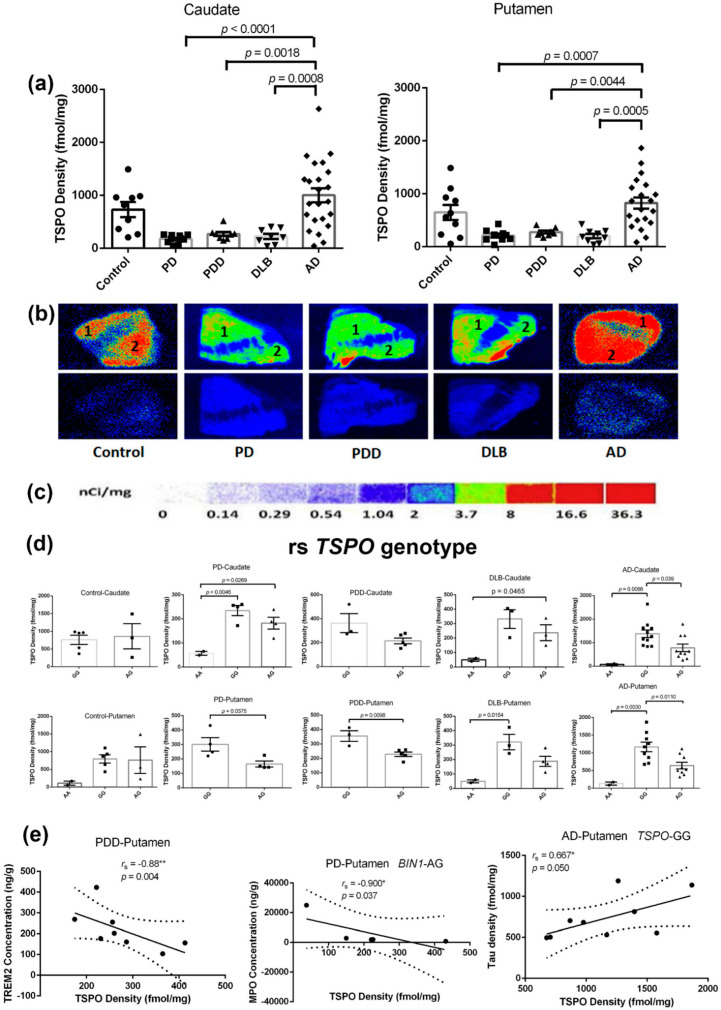
TSPO densities in the caudate and putamen of patients in the disease groups (PD: *n =* 10, PDD: *n =* 8, DLB: *n =* 10, AD: *n =* 27) and age-matched controls (*n =* 10). (**a**): Quantitative analysis of the TSPO densities (fmol/mg) in the caudate and putamen from subjects. Values shown are means ± SEM. Statistical significances between two disease groups are indicated with brackets and corresponding *p*-values. (**b**) Autoradiograms show total binding of 2 nmol/L [^3^H]PBR28 (Panel B top row) and nonspecific binding in the presence of 1 µM PK11195 (Panel B bottom row) in the striatal regions of 5 representative subjects. The numbers 1 and 2 designate the following regions: (1) caudate and (2) putamen. (**c**): [^3^H]Microscale standards (ranging from 0 to 36.3 nCi/mg) were counted alongside samples. (**d**) The *TSPO* rs6971 SNPs impacts the density of TSPO in the caudate and putamen from patients with the disease. Values shown are means ± SEM. Statistical significances between two disease groups are indicated with brackets and corresponding *p*-values. (**e**): Correlations of TSPO vs. TREM2, TSPO vs. MPO, and TSPO vs. Tau. *r*_s_, the Spearman’s rank correlation coefficient. A *p*-value of < 0.05 was considered significant: * indicates *p* < 0.05, ** indicates *p* < 0.01.

TSPO expression is correlated with the extent of microglial activation. We determined the striatal TSPO densities of the disease groups and age-matched controls using quantitative autoradiography. In comparison with controls, lower levels of striatal TSPO binding were obtained in the caudate (PD: −75.7%, PDD: −63.1%, DLB: −69.2%) and putamen (PD: −67.4%, PDD: −57.5%, DLB: −68.9%) samples of LBDs, as shown in Figure 4a, statistically significant results were obtained for the LBDs when compared with AD cases. Conversely, slight increases in TSPO densities of both the caudate (37.0% increase) and the putamen (27.4% increase) of AD cases were found in this study; however, statistical significance was not reached when compared to the controls.

We observed that the *TSPO* rs6971 SNP significantly impacted on the TSPO levels in the caudate and putamen of disease groups (Figure 4d). We genotyped the *TSPO* rs6971 SNP in 65 subjects (PD: *n =* 10, PDD: *n =* 8, DLB: *n =* 10, AD: *n =* 27) and we interrogated differential TSPO levels across this SNP. Significant differences between homozygous GG and heterozygous AG were identified in the putamen of PD, PDD, and AD brains and the caudate of AD cases. No differences were noticed in the controls. TSPO levels in the putamen of homozygous GG carriers were significant higher than heterozygous AG carriers, especially for PD (increased by 82.7%, *p* = 0.0075), PDD (increased by 55.2%, *p* = 0.0098), and AD (increased by 82.5%, *p* = 0.0110). Similar increases were observed in the caudate: TSPO levels in AD patients carrying *TSPO* with homozygous GG genotype showed higher TSPO expressions than heterozygous AG carriers (increased by 77.1%, *p* = 0.0039).

As shown in Figure 4e, Spearman analyses revealed negative correlations between TSPO density and TREM2 concentration in the putamen of PDD brains (*r*_s_ = −0.88, *p* = 0.004), as well as TSPO density and MPO concentration in the putamen of the PD patients carrying *BIN1* with heterozygous AG (*r*_s_ = −0.900, *p* = 0.037). We found obvious positive correlations between TSPO densities and the concentration of tau fibrils in the putamen of AD patients carrying *TSPO* with homozygous GG (*r*_s_ = 0.667, *p* = 0.050), which supports the hypothesis that microglia are involved in the pathological spread of tau [7]. 

### 2.7. Tau Fibrils in the Caudate and Putamen of the Different Groups

**Figure 5:**
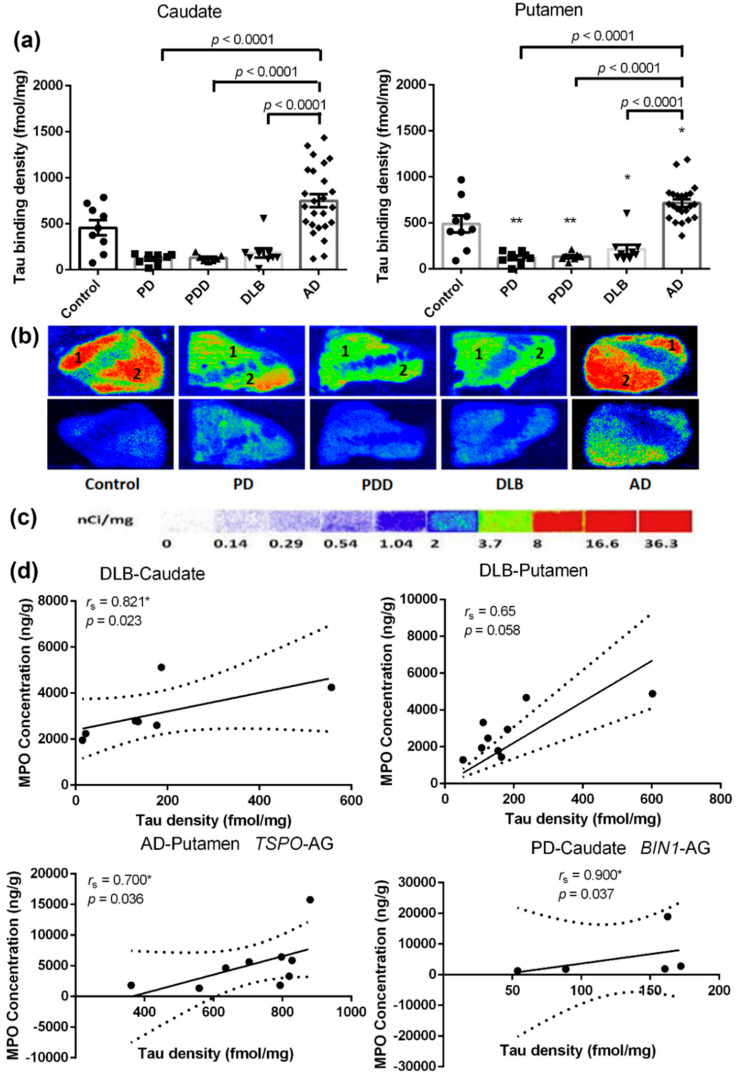
Tau fibrils in the caudate and putamen from patients in disease groups (PD: *n =* 10, PDD: *n =* 8, DLB: *n =* 10, AD: *n =* 27) and age-matched controls (*n =* 10). (**a**): Quantitative analysis of the Tau density (fmol/mg) in the caudate and putamen from subjects. Values shown are means ± SEM. Statistical significances between two disease groups are indicated with brackets and corresponding *p*-values. A *p*-value of < 0.05 was considered significant: * indicates *p* < 0.05, ** indicates *p* < 0.01, vs. controls. (**b**) Autoradiograms show total binding of 2 nmol/L [^3^H]MK6240 (Panel B top row) and nonspecific binding in the presence of 10 µM T807 (Panel B bottom row) in the striatal regions of 5 representative subjects. The numbers 1 and 2 designate the following regions: (1) caudate and (2) putamen. (**c**): [^3^H]Microscale standards (ranging from 0 to 36.3 nCi/mg) were counted alongside samples. (**d**) Correlations between Tau and MPO. *r*_s_, the Spearman’s rank correlation coefficient. A *p*-value of < 0.05 was considered significant: * indicates *p* < 0.05.

We measured tau fibrils of the disease groups and age-matched controls using quantitative autoradiography with [^3^H]MK6240. As shown in Figure 5a, the distribution of tauopathy in the striatal regions was abundant, and no regional differences in binding were found in either the caudate or putamen of the controls (caudate: 457.6 ± 82.6; putamen: 489.3 ± 91.53). Surprisingly, substantial tau fibril reductions with statistical significance were found for the caudate (*p* < 0.0001 vs. AD: PD: −73.8%; PDD: −71.8%; DLB: −60.3%) and putamen of LBDs (PD: −75.2%, *p* = 0.0011; PDD: −72.6%, *p* = 0.0026; DLB: −56.5%, *p* = 0.0176) when compared to controls. In comparison with controls, significant increases of tau densities were found for the putamen of AD cases (increased by 46.4%, *p* = 0.0213). No statistical significance was found in the caudate of AD compared to controls due to large natural variance in these cohorts. 

Interestingly, as shown in Figure 5d, Spearman analyses revealed positive correlations between the concentration of tau fibrils and MPO in the caudate of DLB patients (*r*_s_ = 0.821, *p* = 0.023) and in the putamen of DLB patients (*r*_s_ = 0.65, *p* = 0.058). Furthermore, Spearman analyses uncovered significant positive correlations between the concentration of tau fibrils and MPO in the putamen of the AD patients with the *TSPO* (rs6971) AG genotype (*r*_s_ = 0.700, *p* = 0.036) and the caudate of PD patients with the *BIN1* (rs7561528) AG genotype (*r*_s_ = 0.900, *p* = 0.037). These results shed light on how the misfolded tau is involved in redox imbalance [42].

## 3. Discussion

Interest in microglia and the role of neuroinflammation in neurodegenerative diseases is gaining momentum. Because of the discrepancies between various genetic models used to study the *TSPO* gene in mice, this study has investigated the interactions between oxidative damage, microglia, and tauopathy in humans without losing sight of the importance of single nucleotide polymorphisms.

### 3.1. TREM2 (rs75932628), TSPO (rs6971), and BIN1 (rs7561528) Genotypes of the Study Subjects

A rare missense mutation (rs75932628, p.R47H) in the *TREM2* gene has been reported to crucially increase the risk of developing AD and PD [27,28]. However, we were not able to replicate the positive association between the rs75932628 variant and disease risk in the cohort we studied. The *TSPO* and *BIN1* variants were identified with a statistically non-significant distribution, which might be partially attributed to a small sample size. The rs75932628 variant of the *TREM2* gene was also not identified in a Chinese population with PD, multiple system atrophy (MSA), and amyotrophic lateral sclerosis (ALS) [43,44]. 

A mutation (rs6971) in the *TSPO* gene—the substitution of threonine for alanine at position 147 of *TSPO*—could alter the tertiary structure of the protein [45] and exert several biological effects [46,47]. The *TSPO* (rs6971) SNP has been described as having no impact on cortical *TSPO* mRNA or protein levels in patients with AD [48]. Contrarily, we found a tight linkage between polymorphisms and variance of TSPO levels in the caudate and putamen of PD, PDD, and AD patients. These data indicate that the TSPO level is haplotype dependent, where *TSPO* homozygous GG carriers have significantly higher TSPO levels than heterozygous AG carriers—especially in the putamen (PD 82.7%, PDD 55.2%, and AD 82.5%). Considering no differences were observed in age-matched controls (Figure 4d), we cannot simply ascribe the TSPO level alterations to the different binding affinity induced by the *TSPO* polymorphism (rs6971) [49,50]. Upon microglial activation in the disease pathogenesis, TSPO may experience polymerization leading to the formation of multiple possible binding sites [51]. *TSPO* functional polymorphisms (AG and AA genotypes) may contribute to neuroprotective mechanisms and reduce immune function [52]. Further investigation into understanding the association of *TSPO* gene SNPs and TSPO expression, and these results need to be replicated in a larger population. 

BIN1 could generate tau pathology development, altering tau clearance, and promoting the release of tau enriched extracellular vesicles by microglia [29]. *BIN1*-associated SNPs show the strongest association with AD found and are second only to *Apolipoprotein E* (*APOE*) [53]. We discovered the correlation between the *BIN1* (rs7561528) SNP and expression levels with MPO, TREM2, and PAR. Among patients with LBDs, homozygous GG carriers show higher MPO and TREM2 levels than heterozygous AG carriers, and the inverse was observed for PAR levels. Our results potentially explain the hypothesis that the rs7561528 A allele may be a protective factor against pathology changes and that the G allele of the *BIN1* gene is a possible risk factor for AD [32]. 

### 3.2. Interaction of Reactive Oxygen Species and Microglia in the Caudate and Putamen from Patients with Neurodegenerative Diseases and Age-Matched Controls

Oxidative damage is a hallmark of the aging brains. MPO is a part of the armamentarium of the innate immune system and can be released from vesicles as neutrophils and monocytes [54]. After the release, MPO catalyzes the reactions between hydrogen peroxide (H_2_O_2_) and halides (Cl^−^, Br^−^, I^−^) or pseudohalides (SCN^−^), yielding the corresponding hypohalous acids-potential oxidants [55,56]. MPO binds to microglia resulting in the secretion of ROS and phagocytosis of microglia to remove apoptotic cells and cellular debris, thus promoting additional MPO [57]. In AD, elevated MPO levels have been reported to be localized in senile plaques and some activated microglia of the frontal cortex [58]. However, the more unexpected finding was that no significant MPO alterations could be detected in the striatum (caudate or putamen) of different groups. Consistent with our data, there were no significant differences in MPO in the striatum or cerebellum between PD patients and controls; additionally, the motor cortex of ALS patients does not express higher MPO levels [59]. Thus, these data suggest that brain MPO expression is a generic component of disease or aging-related chronic gliosis rather than a particular etiology [59]. 

It was surprising to find significant positive correlations between the concentration of tau fibrils and MPO in the DLB cases, AD patients with the heterozygous mutation in *TSPO*, and PD patients with the heterozygous mutation in *BIN1*. This finding suggests that misfolded and truncated tau proteins preferentially promote the accumulation of aqueous phase oxidants and free radicals (H_2_O_2_, •OH, and O_2_^•−^), probably activating microglia [42,60]. *TSPO* and *BIN1* heterozygous mutations might, to a certain extent, facilitate ROS accumulation mediated by tau fibril, but this needs to be elucidated in further studies. 

We uncovered positive correlations between MPO levels and PAR concentrations in the putamen of both AD patients with the homozygous GG mutation in *TSPO* and PDD patients with the heterozygous AG mutation in *TSPO*. PAR synthesis was induced by 100 μM H_2_O_2_ in the lymphoblastoid Raji cells [36] and is dependent on the number of damaged DNA sites [61]. We question whether the *TSPO* null mutation AA could play a neuroprotective role against MPO-related damages to DNA. 

In humans, upon recognizing DNA strand breaks, ADP-ribosylation is mainly catalyzed by PAR, using nicotinamide adenine dinucleotide (NAD^+^) as the donor, to link single or multiple ADP-ribose moieties to nuclear targets [62]. This mechanism plays critical roles in DNA repair, replication, and transcription reactions [63]. PAR levels in PD patients have been described as elevated in the substantia nigra and the cerebrospinal fluid (CSF) [64]. However, our previous studies suggest lower/higher DNA adducts levels in the caudate of advanced LBDs/AD [19] and corresponding PAR levels were observed in the same samples studied. The metabolism and amount of dopamine are instrumental in promoting nucleic oxidation as a major source of ROS. The main differences between LBDs and AD lie in the neural substrates with dopamine metabolism being the significant neurochemical difference [65]. *BIN1* heterozygous AG mutation carriers in the PD group showed a higher PAR level in the putamen than homozygous GG carriers, suggesting that the *BIN1* (rs7561528) A allele might be a neuroprotective factor against advanced disease progression. 

A significant negative correlation was found between the PAR concentrations and TREM2 expressions in the caudate of AD patients (*r*_s_ = −0.542, *p* = 0.020), most likely because one of the molecular switches, PARylation, may be responsible for transiting microglia towards the inflammatory phenotype [24,37,66].

TREM2 is a transmembrane receptor of the immunoglobulin superfamily predominantly expressed on microglia in the brain [8]. TREM2 expression on microglia cells correlates with a specific activated microglial phenotype and helps to switch between the classic inflammatory M1 and immunosuppressive M2 phenotypes [67]. Different stages of disease-associated-microglia were regulated independently/dependently by TREM2, indicating high microglial heterogeneity in individual patients during the disease progression [41]. Correlations between TREM2 levels and disease progression/PD stage strongly supported this hypothesis (Figure 3d, Figure S3 and Figure S4). Several studies have shown higher TREM2 levels in the midbrain of PD mice [25] and TREM2 highly expressed in microglia of AD brains or AD model mice [38,68]. We observed trends of increasing and decreasing TREM2 expressions in the caudate and putamen of the LBDs without statistical significance. Compared to decreased TSPO levels in the caudate of patients with LBDs, the increase of TREM2 in this brain region seems to be a compensatory attempt of TREM2 to attenuate the excessive microglial over-activation or ameliorate non-responsive microglial types [25]. 

Further evidence for this explanation can be found in the negative correlation between TREM2 concentrations and TSPO densities in the putamen of the PDD patients (*r*_s_ = −0.88, *p* = 0.004). DLB patients carrying the homozygous *BIN1* (rs7561528) GG mutation showed remarkably higher TREM2 levels in the caudate than the heterozygous AG carriers, suggesting a more compensatory mechanism of the GG mutation-a risk factor in AD pathogenesis. The positive correlation between TREM2 levels and 8-oxo-dG concentration in the caudate from DLB cases (*r*_s_ = 0.881, *p* = 0.004, Figure S1) made us curious about whether TREM2 can recognize changes in the environment or send a signal to keep microglia ready to go. Besides, a negative correlation between the concentration of TREM2 and tau fibrils was identified in the putamen of DLB cases, and CSF soluble TREM2 has been clarified as highly associated with total tau and NFT densities (phosphorylated tau) [69]. The possible mechanism of TREM2 associated tau pathology is disturbing the contact between microglia and neurons and affecting the pathological spread of tau [70].

Yield from dopamine metabolism, free radicals might induce microglia activation as indirectly indicated by the positive correlation between dopamine concentrations and TSPO densities in the putamen of PD patients (*r*_s_ = 0.898, *p* = 0.001, Figure S2). Additionally, the activated microglia, in turn, might cause dopaminergic neuronal death and damage to microglia and consequently release additional free radicals [71,72]. This hypothesis was supported by the negative correlation between MPO concentrations and TSPO densities in the putamen of PD patients carrying the heterozygous *BIN1* (rs7561528) AG mutation (*r*_s_ = −0.900, *p* = 0.037).

### 3.3. Microglia Implicated in Tauopathy in the Caudate and Putamen from Patients with Neurodegenerative Diseases and Age-Matched Controls

Increased microglial activation has been described in postmortem brain samples of AD [73,74]. The activated microglia aggregate with dopaminergic neurons in postmortem PD patient brains, suggesting microglia was involved in the pathological progress of dopaminergic neurons [75]. Our previous data shows a significant decrease in TSPO densities of the substantia nigra in AD and DLB brains compared to that of controls. It is explained by the possible microglia dystrophy in advanced neurodegeneration [13]. However, this is not unique; we discovered similar alterations in different cohorts—significant reductions of TSPO density in the striatum (caudate and putamen) of LBDs and a slight increase in TSPO density in the striatum of AD cases. Once over-activated microglia reaches an exhausting phase, it has been traditionally believed that they cannot escape their broad defects in energy metabolism, leading to a diminished immune response, namely dystrophic or senescent microglia [76,77]. 

Microglial burden correlates with tau burden in the main pathologically afflicted areas [78,79]. This hypothesis is further supported by a positive correlation between TSPO density and tau density in the putamen of AD patients carrying the homozygous *TSPO* GG mutation (*r*_s_ = 0.667, *p* = 0.050). Parallel changes in TSPO density and tau fibrils in the caudate and putamen of disease groups suggest that microglia may be associated with tau pathology. In comparison with the controls, we observed significant decreases and increases of tau fibrils in both the caudate and putamen of both LBDs and AD. Expression of tauopathy in the brains of PD and PDD might present very restricted patterns of distribution. It may be limited to dopaminergic neurons of the nigrostriatal region, a stark contrast to AD, where tauopathy is more universally found in various brain areas [6,80]. Microglia may be essential for tau propagation via the release of exosomes [7]. Thus, changing microglial phenotypes might contribute to tau pathology and propagation, partially resulting in altered tau proteostasis. 

The omission of other dystrophic/senescent microglia biomarkers, microglial mutation genes, a limited sample size, and significant dispersions in data are limitations to this study. We were currently inspired to continue single-nuclei RNA sequence research to remedy these limitations and address other questions that arise in this context.

## 4. Materials and Methods 

### 4.1. Ethics Statement

According to local ethical committee procedures, patients provided written consent before cognitive impairment or the next of kin provided consent antemortem or postmortem (Washington University Institutional Review Board, Washington University School of Medicine, St Louis, MO, USA). The use of tissue for autoradiography and biochemistry research was approved by the Charles F. and Joanne Knight Alzheimer’s Disease Research Center and Movement Disorders Center Leadership Committees (Ethics approval reference number: T1705).

### 4.2. Radioligands 

[^3^H]PBR28 (80 Ci/mmol, CAS Number: 253307-72-1) was purchased from American Radio Label (St Louis, MO, USA) and [^3^H]MK6240 (21.5 Ci/mmol, CAS Number: 1841078-87-2) was purchased from Vitrax (Placentia, CA, USA).

### 4.3. Subjects

Clinically and neuropathologically well-clarified human brain tissues were obtained from the Knight ADRC and the MDC Brain Bank at Washington University School of Medicine. The tissues collected were as follows: 10 PD (7 males, 3 females) aged 69–87 (mean: 78 ± 2) years at death, 8 PDD (7 males, 1 female) aged 66–87 (mean: 77 ± 3) years at death, 10 DLB (5 males, 5 females) aged 69–89 (mean: 81 ± 2) years at death, 27 AD (13 males, 14 females) aged 62–94 (mean: 82 ± 2) years at death, and 10 age-matched healthy control cases (6 males, 4 females) aged 72–93 (mean: 83 ± 2) years at death. The arbitrary clinical division between DLB and PDD was made using the criteria of McKeith et al. [81,82]. Dementia level was evaluated by Clinical Dementia Rating (CDR) [83]. Based on the CDR criteria for diagnosing dementia in PD, participants with a CDR ≥ 1 were taken. AD pathological changes were estimated using Braak staging [84]. Braak stages of amyloid-beta deposition use letters: (A) the initial deposits in the basal neocortex, (B) deposits that extend into the adjacent areas of the neocortex, and (C) heavy deposition throughout the entire cortex. Stages of neurofibrillary pathology represent transentorhinal (I-II), limbic (III-IV), and neocortical (V and VI). The average age and postmortem interval time did not significantly differ across groups. All the AD cases show neurofibrillary tangles (NFTs) (V: 13 cases; VI: 14 cases) and are significantly different from those of the age-matched control cases (Average amyloid-beta: A; NFTs: II). Additionally, all LBDs showed NFTs. The clinical information and pathological features are summarized in Table 1.

### 4.4. Tissue Collection 

Brains were harvested at the time of autopsy, and the right hemisphere was coronally sectioned and snap-frozen using Teflon-coated aluminum plates cooled in liquid nitrogen vapor. Then, tissue blocks were placed in airtight zip-lock plastic bags and preserved at −80 °C until used. Frozen coronal sections (20 µm) were cut using a Cryostat Microm and mounted immediately on Superfrost Plus glass slides (Fisher Scientific, Pittsburgh, 1255015, PA, USA) for autoradiography study. Striatal sub-areas, the caudate and putamen, were carefully dissected using a scalp from adjacent cryosections and tested for the biochemistry and genetic assays separately. 

### 4.5. Quantitative Analysis of Myeloperoxidase (MPO)

The levels of MPO in the caudate and putamen of the study brains were measured using a Myeloperoxidase Human ELISA Kit (Abcam, ab119605, Cambridge, MA, USA) according to the protocol provided by the manufacturer. First, samples (100 mg) were homogenized in 5 mL lysis buffer (Fisher Scientific, 895347, Pittsburgh, PA, USA), and the mixtures were centrifuged for 10 min (10,000× *g*, 4 °C). Next, the supernatant was collected and diluted (1:10) for use with the MPO assay kit. Each prepared sample was detected in repeat. Known standards were added to the same assay in triplicate for accurate quantification. The detection range for MPO was 312–20,000 pg/mL, and the sensitivity was <10 pg/mL. All data were calibrated using a standard curve with a regression coefficient *r*^2^ > 0.99.

### 4.6. Quantitative Analysis of Poly (ADP-Ribose) (PAR)

The concentrations of PAR in the caudate and putamen from study brains were detected with the commercially available PAR ELISA kit (Cell Biolabs, Inc., XDN–5114, San Diego, CA, USA) according to the user’s manual provided by the manufacturer. First, samples (100 mg) were homogenized in 1 mL RIPA buffer (Fisher Scientific, AKR-190, Pittsburgh, PA, USA) containing 1X PARP Inhibitor (1mg/mL 3-AB, Cell Biolabs, Inc., XDN–5114, San Diego, CA, USA), and the mixtures were centrifuged for 10 min at 10,000× *g* at 4 °C. Next, the supernatant was collected, and SDS was added to a final concentration of 1% and boiled (100 °C) for 5 min. Finally, the solution was snap-cooled on the ice and centrifuged at 10,000× *g* for 5 min. The resulting supernatant was used with the PAR assay kit directly. Each prepared sample was added to the assay in repeat. Known standards were added to the same assay in triplicate to perform accurate quantification. All data were calibrated from a standard curve with *r*^2^ > 0.98.

### 4.7. Quantitative Analysis of Triggering Receptor Expressed on Myeloid Cell 2 (TREM2)

The expression of TREM2 in the caudate and putamen of snap-frozen brains was assessed using a TREM2 Human ELISA kit (Biomatik, EKU07882, Wilmington, DL, USA) according to the manufacturer’s instructions. 100 mg samples were homogenized in 5 mL lysis buffer (Fisher Scientific, 895347, Pittsburgh, PA, USA), and the mixtures were centrifuged for 10 min (10,000× *g*, 4 °C). The supernatant was collected and diluted (1:5) for use with the TREM2 assay kit. Known standards were added in triplicate, and each sample was detected in repeat in the same assay. The detection range for MPO was 62.5–4000 pg/mL and the sensitivity was <26.2 pg/mL. All data were calculated using a standard curve with *r*^2^ > 0.99.

### 4.8. Autoradiography

#### 4.8.1. Quantification of Total Radioactivity

Dried slides were made by covering the free side with copper foil tape. Then slides were placed into a gas chamber containing a balance of argon and triethylamine (Sigma-Aldrich, BP616-500, St. Louis, MO, USA) as part of a gaseous detector system, Beta Imager 2000Z Digital Beta Imaging System (Biospace, Nesles la Vallée, France) with a sensitivity limit of 0.07dpm/mm^2^. When a homogenous state was achieved, further exposure for 20 h yielded high-quality images. A [^3^H]microscale with known radioactivity (ranging from 0 to 36.3 nCi/mg) was counted with each section and used to establish a standard curve with a correlation coefficient of *r*^2^ > 0.99. Quantitative analysis was accomplished with the program Beta-Vision Plus (BioSpace, Nesles la Vallée, France) for each region of interest.

#### 4.8.2. 18-kDa Translocator Protein (TSPO) Binding

TSPO binding sites were labeled with [^3^H]PBR28. Brain sections were incubated at room temperature (RT) for 30 min in a buffer solution containing 2 nmol/L [^3^H]PBR28 (American Radio Label, ART2270, MO, USA) and followed by rinses (1 min × 5 times). Nonspecific binding was determined using the adjacent sections in 1 µmol/L PK11195 (Bio-Techne Corporation, 0670, Minneapolis, MN, USA) as previously described [13].

#### 4.8.3. Tau Fibrils Binding

Tau fibrils binding sites were labeled with [^3^H]MK6240. Brain sections were incubated at RT for 30 min in a buffer solution containing 2 nmol/L [^3^H]MK6240 (Vitrax, VT298, Placentia, CA, USA) and followed by rinses (1 min × 5 times). Nonspecific binding was determined using the adjacent sections in the presence of 10 µmol/L T807 (MedChemExpress, HY-101184, Monmouth Junction, NJ, USA). 

### 4.9. Genotype

According to the manufacturer’s instruction, total DNA in the striatum of study brains was extracted using the Qiagen QIAamp DNA Mini Kit (Qiagen, Valencia, 51304, CA, USA). A NanoDrop 1000 spectrophotometer (Thermo Fisher, Pittsburgh, PA, USA, RRID: SCR_016517) was utilized to measure DNA integrity and purity. Samples interrogated rs6971 (*TSPO*), rs75932628 (*TREM2*), and rs7561528 (*BIN1*) separately using Taqman SNP Assays (ThermoFisher Scientific, Grand Island, NY, USA). A master mix of 0.5 uL of 40× Taqman SNP Assay, 40 ng of DNA from each sample, and 10 uL of Taqman Universal PCR was used to amplify the specific allele for each specimen in a total reaction volume of 20 uL. The cycling program consisted of 10 min at 95 °C, followed by 40 cycles of 95 °C for 15 sec and then 60 °C for 1 min on a CFX96 thermocycler (Biorad). The calls were analyzed and viewed using the Bio-Rad CFX Manager software.

### 4.10. Statistical Analysis

Data were expressed as means ± SEM. The statistical analyses were performed using all data without any further normalization. A one-way analysis of variance (ANOVA) with Tukey’s post-hoc-tests was used to estimate the overall significance of normally distributed datasets, and a Kruskal-Wallis ANOVA with Dunn’s multiple comparison test was run if the dataset was not normally distributed. A student’s unpaired t-test was used to assess the difference between groups. Spearman’s correlation coefficient (rs) was calculated to verify the strength of the correlation between continuous variables. *TSPO* (rs6971) and *BIN1* (rs7561528) genotype distributions (AA vs. AG vs. GG) were compared between disease and control groups with a Chi-square test. Statistical analyses were carried out using GraphPad Prism 6.0 (RRID: SCR_002798) for Windows and IBM SPSS Statistics version 23 (RRID: SCR_002865). In all cases, *p* < 0.05 was considered statistically significant. No randomization, blinding, or sample size calculations were performed during experimentation or statistical analyses. Error bars on the scatter dot plots represent standard error of the mean. 

## 5. Conclusions

This study discusses how ROS interacts with microglia in the striatum of patients with advanced neurodegenerative diseases based on the region-specific alteration in levels of microglial neurobiological phenotypes: MPO, PAR, and TREM2. The parallel changes of TSPO densities and tau fibrils provide a better understanding of how microglia are implicated in tauopathy. This study is the first to establish genotype–phenotype relationships to some extent, address how some SNPs relate to specific aspects of microglial function and lead to tau proteostasis disturbance. Nevertheless, even if some gray areas persist regarding the mechanisms and interactions between microglia and tauopathy, future directions for study are already emerging with this framework.

## Figures and Tables

**Table 1:** Baseline information and clinical features of the study subjects. PMI: Postmortem Interval; Braak neurofibrillary tangles (NFT) stage: Braak neurofibrillary tangle stage; Braak Aβ stage: Braak amyloid-beta plaque stage. * Indicates *p* < 0.05 vs. the controls.

	Control	PD	PDD	DLB	AD	P
Participants	10	10	8	10	27	
Male/Female	6/4	7/3	7/1	5/5	13/14	NA
Age	83 ± 2	78 ± 2	77 ± 3	81 ± 2	82 ± 2	NA
PMI (h)	18.8 ± 5	16.0 ± 3.2	10.8 ± 1.6	18.0 ± 3.7	10.6 ± 1	NA
Brain weight (g)	1326 ± 60	1299 ± 40	1346 ± 41	1273 ± 38	1127 ± 47	NA
Onset		65 ± 3	60 ± 3	66 ± 4	71 ± 2	NA
Progression		14 ± 1	16 ± 3	14 ± 3	10 ± 1	NA
Braak NFT stage	Stage 0:1	Stage I:5	Stage I:3	Stage I:7	Stage V:13	*
	Stage I:2	Stage II:2	Stage II:2	Stage II:2	stage VI:14	
	Stage II:4	Stage III:3	Stage III:3	Stage V:1		
	Stage III:3					
Braak Aβ stage	All normal	Normal:3	Normal:1	Normal:1	All stage C	NA
		Stage A:1	Stage A:2	Stage A:1		
		Stage B:2	Stage B:1	Stage B:2		
		Stage C:5	Stage C:4	Stage C:6		
L-Dopa response		Yes: 9	Yes: 6	Yes: 9		
		Modest:1	Modest:2	Modest:1		

**Table 2:** Detection of *TSPO* and *BIN1* single nucleotide polymorphisms (SNPs) genotype (AA, GG, and AG) in 65 neurodegenerative disease patients with the absence of *TREM2* gene variants. There are two samples, from the Parkinson’s disease (PD) and dementia with Lewy bodies (DLB) groups, that clustered oddly for the *BIN1* (rs7561528).

Group	TSPO (rs6971) Genotype						BIN1 (rs7561528) Genotype					
	AA		GG		AG		AA		GG		AG	
Controls	2	20%	5	50%	3	30%			5	50%	5	50%
PD	2	20%	4	40%	4	40%			4	44.4%	5	55.6%
PDD			3	37.5%	5	62.5%	1	12.5%	4	50%	3	37.5%
DLB	2	20%	4	40%	4	40%	1	11.1%	5	55.6%	3	33.3%
AD	2	7.4%	12	44.4%	13	48.2%	2	7.4%	11	40.7%	14	51.9%
Chi-square, df *p* value	3.417, 8 0.9055						1.097, 4 0.8947					

## Supplementary Materials

The following are available online at https://www.mdpi.com/1422-0067/21/17/6047/s1, Figure S1. Correlation between TREM2 levels and 8-oxo-dG concentration in the caudate from DLB cases. *r*_s_, the Spearman’s rank correlation coefficient. Figure S2. Correlation between dopamine concentrations and TSPO densities in the putamen of PD patients. *r*_s_, the Spearman’s rank correlation coefficient. Figure S3. Correlation between TREM2 levels in the putamen from PDD cases and Disease Progression. *r*_s_, the Spearman’s rank correlation coefficient. Figure S4. Correlation between TREM2 levels in the putamen from LBDs and PD stage. *r*_s_, the Spearman’s rank correlation coefficient.

## Abbreviations

AD	Alzheimer’s disease
PD	Parkinson’s disease
MPO	Myeloperoxidase
PAR	Poly (ADP-Ribose)
TREM2	Triggering receptors expressed on myeloid cell 2
ELISA	Enzyme-linked immunosorbent assay
TSPO	Translocator protein
DLB	Dementia with Lewy body
PDD	Parkinson’s disease dementia
LBDs	Lewy body diseases
BIN1	Bridging integrator 1
SNP	Single-nucleotide polymorphism
MAPT	Microtubule-associated protein tau
NFTs	Neurofibrillary tangles
PHFs	Paired helical filaments
CNS	Central nervous system
MPTP	1-Methyl-4-phenyl-1, 2,3,6-tetrahydropyridine
ERK	Extracellular signal-regulated kinase
SNc	Substantia nigra pars compacta
ROS	Reactive oxygen species
PARP	Poly (ADP-ribose) polymerase
FTD	Frontotemporal dementia
RT	Room temperature
ANOVA	Analysis of variance
MSA	Multiple system atrophy
ALS	Amyotrophic lateral sclerosis
APOE	Apolipoprotein E
NAD^+^	Nicotinamide adenine dinucleotide
CSF	Cerebrospinal fluid
