# Supplementary material for: Microglia Implicated in Tauopathy in the Striatum of Neurodegenerative Disease Patients from Genotype to Phenotype
Source: Int J Mol Sci. 2020 Aug 22;21(17):6047. doi: 10.3390/ijms21176047 (PMC7503242; doi:10.3390/ijms21176047)
Supplement: Supplementary file 1 [file ijms-21-06047-s001.zip › ijms-910024-supplementary.docx]

**Supplementary Materials**


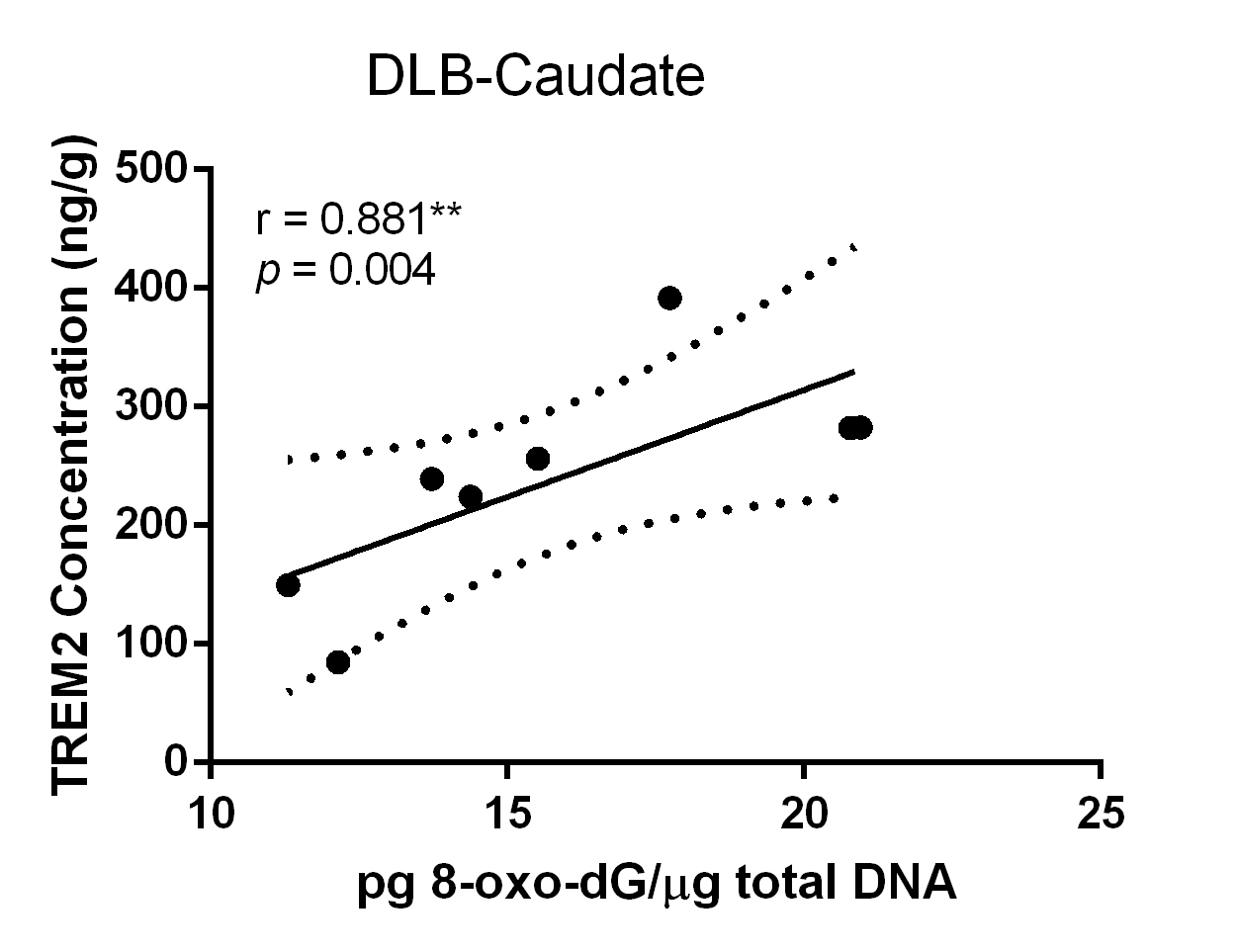


**Figure S1.** Correlation between TREM2 levels and 8-oxo-dG concentration in the caudate from DLB cases. *r*_s_, the Spearman’s rank correlation coefficient. A *p*-value of < 0.05 was considered significant: ** indicates *p* < 0.01.


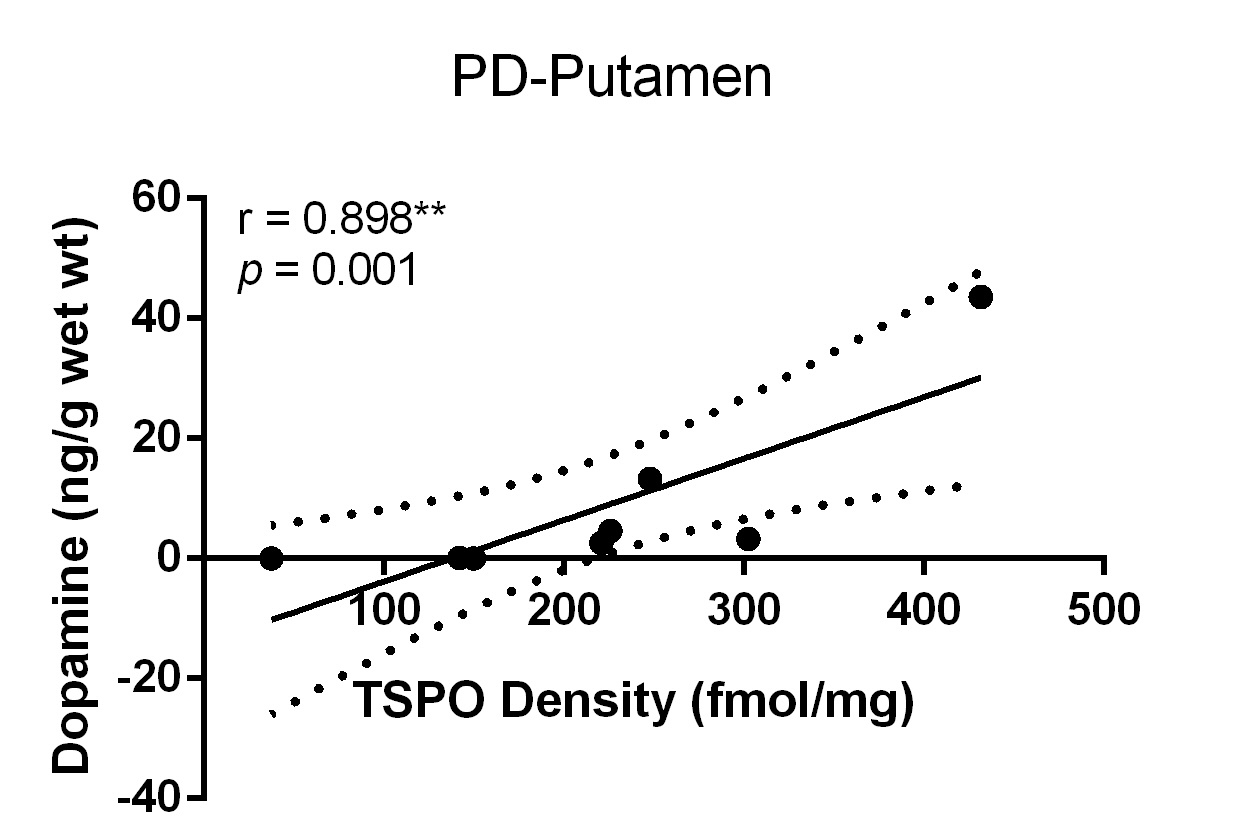


**Figure S2**. Correlation between dopamine concentrations and TSPO densities in the putamen of PD patients. *r*_s_, the Spearman’s rank correlation coefficient. A *p*-value of < 0.05 was considered significant: ** indicates *p* < 0.01.


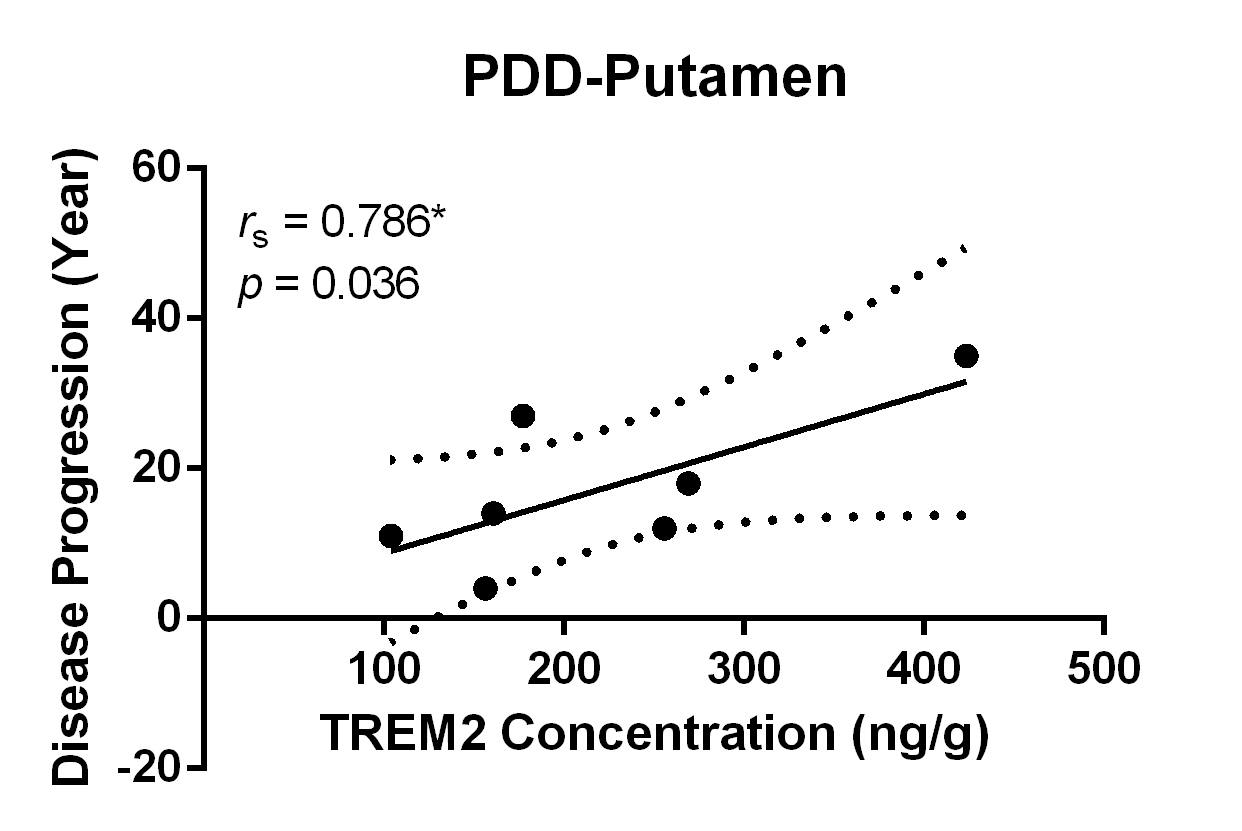


**Figure S3**. Correlation between TREM2 levels in the putamen from PDD cases and Disease Progression. *r*_s_, the Spearman’s rank correlation coefficient. A *p*-value of < 0.05 was considered significant: * indicates *p* < 0.05.


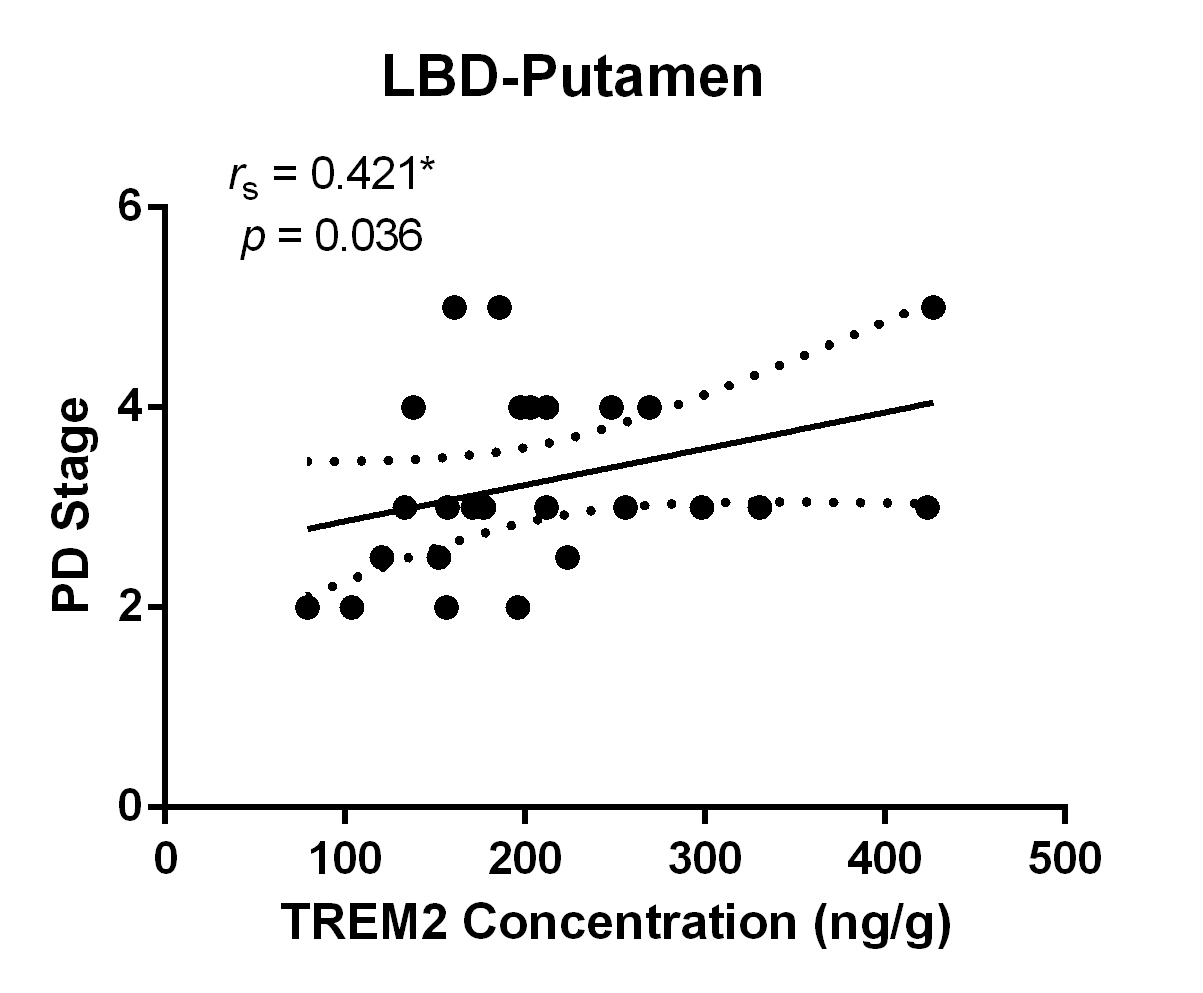


**Figure S4**. Correlation between TREM2 levels in the putamen from LBD cases and PD stage. *r*_s_, the Spearman’s rank correlation coefficient. A *p*-value of < 0.05 was considered significant: * indicates *p* < 0.05.
